# Efficacy and effectiveness of COVID-19 vaccines against SARS-CoV-2 infection: interim results of a living systematic review, 1 January to 14 May 2021

**DOI:** 10.2807/1560-7917.ES.2021.26.28.2100563

**Published:** 2021-07-15

**Authors:** Thomas Harder, Judith Koch, Sabine Vygen-Bonnet, Wiebe Külper-Schiek, Antonia Pilic, Sarah Reda, Stefan Scholz, Ole Wichmann

**Affiliations:** 1 Robert Koch Institute, Berlin, Germany

**Keywords:** SARS-CoV-2, systematic review, vaccine effectiveness, vaccination, COVID-19

## Abstract

Evidence on COVID-19 vaccine efficacy/effectiveness (VE) in preventing asymptomatic SARS-CoV-2 infections is needed to guide public health recommendations for vaccinated people. We report interim results of a living systematic review. We identified a total of 30 studies that investigated VE against symptomatic and/or asymptomatic infection. In fully vaccinated individuals, VE against symptomatic and asymptomatic infections was 80–90% in nearly all studies. Fully vaccinated persons are less likely to become infected and contribute to transmission.

Vaccination against severe acute respiratory syndrome coronavirus 2 (SARS-CoV-2) infection plays a key role in the containment of the coronavirus disease (COVID-19) pandemic. All vaccines approved by the European Medical Agency (EMA) at the time of writing demonstrated high vaccine efficacy/effectiveness (VE) against severe COVID-19. With vaccination programmes being implemented in most European countries, it becomes urgent to assess the extent to which these vaccines are also able to prevent symptomatic and asymptomatic infections to guide public health recommendations and develop strategies for fully vaccinated people. 

In December 2020, the Robert Koch Institute (RKI), in collaboration with the National Immunisation Technical Advisory Groups (NITAGs) network coordinated by the European Centre for Disease Prevention and Control (ECDC) initiated a living systematic review on the VE and safety of European Union (EU)-licensed COVID-19 vaccines (PROSPERO registration: CRD42020208935). In this paper, only efficacy and effectiveness data but not those on safety will be covered. In detail, we report the interim results of the review focusing on two research questions:

- What is the efficacy/effectiveness of COVID-19 vaccines in preventing SARS-CoV-2 infections (irrespective of whether those infected were symptomatic or asymptomatic)?
- What is the efficacy/effectiveness of COVID-19 vaccines in preventing asymptomatic SARS-CoV-2 infections?

## Literature search 

This living systematic review follows the recommendations of the Preferred Reporting Items for Systematic Review and Meta-Analysis (PRISMA) guideline (Supplement Part S1). Monthly searches were done and results were immediately incorporated into the evidence base. We considered studies of any design as long as they had a comparison group that investigated VE against SARS-CoV-2 infection and/or asymptomatic SARS-CoV-2 infection after vaccination with an EMA-approved COVID-19 vaccine (see Supplement Part S2 for complete population intervention comparison outcomes (PICO) questions). No restrictions were made regarding publication language or status. The review started on 1 January 2021. The end date of this interim analysis was 14 May 2021.

We used an internal COVID-19 literature database constructed by the RKI library to search for relevant studies. This database covers PubMed, Embase (including Medline) and the preprint servers ArRvix, BioRxiv, ChemRxiv, MedRxiv, Preprints.org, ResearchSquare and Social Science Research Network (SSRN) (see Supplement Part S3 for search strategy). In addition, we hand-searched the websites of the ECDC, the United States (US) Centers for Disease Control and Prevention, the Public Health Agency of Canada and Public Health England for additional studies and reports. Potentially relevant publications were screened at title/abstract and full-text level by two independent investigators for eligibility. Disagreements were resolved by discussion. From the identified studies, data were extracted as described in the PROSPERO protocol and summarised in tabular form. For randomised controlled trials (RCTs), risk of bias was assessed using the Cochrane Risk of Bias tool-2 (RoB-2) [1]. To non-randomised studies, ROBINS-I was applied [2].

Due to heterogeneity of study design, time point of analysis, vaccine used, population and settings, we did not perform a meta-analysis.

## Study screening 

By 14 May 2021 (the date of last search), we identified and screened a total of 4,870 entries. After screening 204 full-text articles, 30 studies were included (Figure) [3-32]. Twenty-six studies reported on infections (irrespective of whether they were symptomatic or not), including three studies that reported both outcomes, and four additional studies reported exclusively on asymptomatic infections. 

**Figure:**
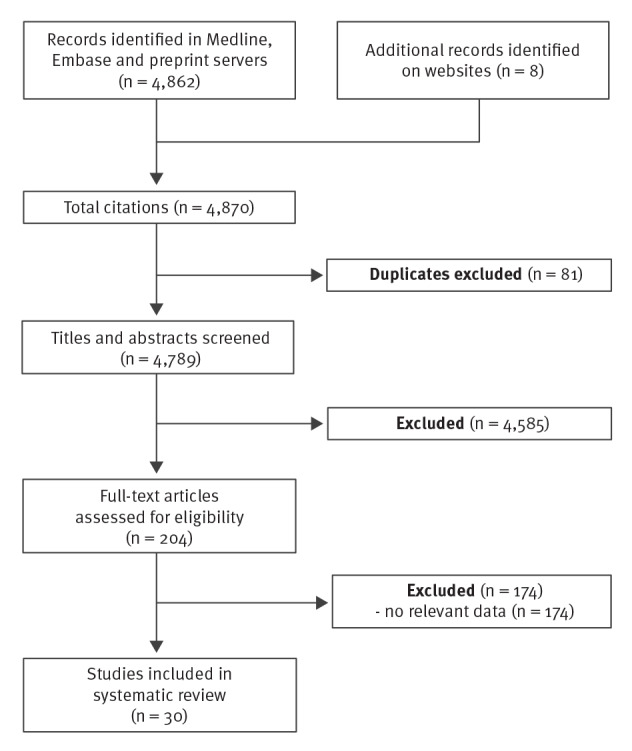
PRISMA flowchart of the living systematic review on efficacy and effectiveness of COVID-19 vaccines against SARS-CoV-2 infection

COVID-19: coronavirus disease; SARS-CoV-2: severe acute respiratory syndrome coronavirus 2; PRISMA: Preferred Reporting Items for Systematic Review and Meta-Analysis.

Date of last search: 14 May 2021.

### Prevention of infection

Of the 30 studies, 26 investigated the efficacy/effectiveness of COVID-19 vaccines in preventing SARS-CoV-2 infections, based on reports of number of symptomatic and asymptomatic PCR-positive individuals [3-18,22-31] (Table 1). Studies were conducted in eight different countries (Denmark (n=1), Israel (n=4), Italy (n=1), Qatar (n=1), Spain (n=2), Sweden (n=1), UK (n=8), US (n=7)) and one study was multi-centric [22]. They included between 463 and 2,183,000 participants aged 16–99 years. Two studies were RCTs, 19 studies had a cohort design and five were case–control studies, including two with test-negative design. In 12 studies, the effectiveness of Comirnaty (BionTech, Mainz, Germany/Pfizer, Puurs, Belgium) was evaluated. Two studies investigated COVID-19 Vaccine Janssen (Janssen-Cilag International, Beerse, Belgium), one study studied Vaxzevria (AstraZeneca/Oxford, Oxford, United Kingdom (UK)), and 11 studies investigated more than one vaccine.

**Table 1:** Efficacy and effectiveness of COVID-19 vaccines against SARS-CoV-2 infection (symptomatic and asymptomatic), 1 January–14 May 2021 (n = 26)

Study	Country	Study design	Study population (n)	Age	Circulating variant	Vaccine	Time point of analysis after vaccine dose	Adjusted vaccine efficacy/effectiveness (95% CI)	
								After dose 1	After dose 2
Abu-Raddad [23]; 10 May 2021	Qatar	Case–control study (test-negative design)	General population (cases: n = 35,979; controls: n = 35,979)	Adults (median: 32 years)	Alpha (B.1.1.7); Beta (B.1.351)	Comirnaty	After dose 1; ≥ 14 days after dose 2	Alpha: 29.5% (22.9–35.5) Beta: 16.9% (10.4–23.0)	Alpha: 89.5% (85.9–92.3) Beta: 75.0% (70.5–78.9)
Amit [3]; 18 Feb 2021	Israel	Retrospective cohort study	HCW (n = 9,109)	Adults	Not reported	Comirnaty	15–21 days after dose 1; 22–28 days after dose 1; 6 days after dose 2	65% (43–68) 75% (72–84) (including some persons vaccinated with two doses)	Not reported
Andrejko [4]; 10 Apr 2021^a^	US	Case–control study (test-negative design)	Population-based (325 cases; 320 controls)	≥ 18 years	Not reported	Comirnaty, Moderna	8–14 days after dose 1 or dose 2; ≥ 15 days after dose 1 or 2	66.3% (− 68.7–93.3) 58.9% (− 9.7–84.5%)	78.4% (23.2–94.3) 85.7% (67.2–93.9)
Björk [24]; 21 Apr 2021	Sweden	Cohort study	Population-based (n = 805,741)	18–64 years	Not reported	Comirnaty	> 14 days after dose 1; > 7 days after dose 2	42% (14–63)	86% (72–94)
Britton [5]; 19 Mar 2021^a^	US	Retrospective cohort study	LTCF inhabitants (n = 463)	Not reported	Not reported	Comirnaty	> 14 days after dose 1	63% (33–79)	Not reported
Chodick [6]; 29 Jan 2021^a^	Israel	Retrospective cohort study	Insurance members (n = 503,875)	≥ 16 years, mean: 59.7 years (SD: 14.7)	Not reported	Comirnaty	> 13 days after dose 1	51.4% (− 7.2–78)	Not reported
Corchado-Garcia [25]; 30 Apr 2021	US	Retrospective cohort study	Mayo Clinics health system records (vaccinated: 2,195; unvaccinated: 21,950)	≥ 18 years	Not reported	COVID-19 vaccine Janssen	> 15 days after dose	76.7% (30.3–95.3)	Not applicable
Dagan [7]; 15 Apr 2021	Israel	Matched case–control study	Insurance members (n = 1,193,236)	≥ 16 years, median: 45 years	80% Alpha (B.1.1.7)	Comirnaty	≥ 7 days after dose 2	46% (40–51)	92% (88–95)
EMA Assessment report COVID-19 Vaccine Janssen [22]; 11 Mar 2021	Multi-centre (incl. US, Brazil, South Africa)	RCT (phase 3-licensure trial)	Vaccine group (n = 19,306); placebo group (n = 19,178)	≥ 18 years	Beta (B.1.351); Zeta (P.2); D614G-carrying ‘WT/ref’	COVID-19 vaccine Janssen	14 days after vaccination	67.2% (56.86–75.26)	Not applicable
Emary [8]; 30 Mar 2021	UK	RCT	Randomised population, 66% working in health and social care settings (vaccinated n = 4,244; control: n = 4,290)	≥ 18 years	Alpha (B.1.1.7); Non-Alpha	Vaxzevria	≥ 15 days after dose 2	Not reported	Non-Alpha: 77.3% (65.4–85.0); Alpha: 61.7% (36.7–76.9)
Fabiani [26]; 29 Apr 2021	Italy	Retrospective cohort study	HCW (n = 6,423)	Mean: 47.1 years (SD: 10.8 years)	Not reported	Comirnaty	14–21 days after dose 1; ≥ 7 days after dose 2	84% (40–96)	95% (62–99)
Glampson [9]; 10 Apr 2021^a^	UK	Retrospective cohort study	Population-based (n = 2,183,939; (n = 389,587 vaccinated)	≥ 16 years	Alpha (B.1.1.7)	Comirnaty; Vaxzevria	28 days after dose 1	Vaxzevria: 74% (HR: 0.26 (0.19–0.35) Comirnaty: 78% (HR: 0.22 (0.18–0.27))	Not reported
Guijarro [10]; 26 Mar 2021^a^	Spain	Cohort study	HCW: n = 2,590 (cf.d with average population: n = 170,513)	Not reported	Not reported	Comirnaty	2–4 weeks after dose 1; 7 days after dose 2	63% incidence reduction (cf.d with average population)	99% incidence reduction (cf.d with average population)
Haas [11]; 24 Mar 2021	Israel	Cohort study	Surveillance data (national); n = 202,684 SARS-CoV-2 infections; n = 102,012 non-vaccinated	≥ 15 years	94.5% Alpha (B.1.1.7)	Comirnaty	≥ 7 days after dose 2; ≥ 14 days after dose 2	Not reported	95.3% (94.9–95.7) 96.5% (96.3–96.8)
Hall [12]; 22 Feb 2021	UK	Cohort study	HCW without previous SARS-CoV-2 infection (n = 23,324)	Median: 46.1 years (IQR: 36.0–54.1)	Alpha (B.1.1.7)	Comirnaty	21 days after dose 1; 7 days after dose 2	72% (58–86)	86% (76–97)
Lumley [13]; 12 Mar 2021	UK	Cohort study	HCW (n = 13,109)	Median: 39 years (range: 30–50)	Alpha (B.1.1.7 )	Comirnaty; Vaxzevria	> 14 days after dose 1 and dose 2	64% (aIRR = 0.36 (0.26–0.50))	90% (aIRR = 0.10 (0.02–0.38))
Mason [27]; 22 Apr 2021^a^	UK	Matched case–control study	Population (n = 170,226)	80–83 years	Alpha (B.1.1.7)	Comirnaty	21 to 27 days after dose 1; 35–41 days after dose 1 and 7 days after dose 2	55.2% (40.8 - 66.8) 70.1% (55.1–80.1) (including persons vaccinated with two doses	Not reported
Menni [28]; 27 Apr 2021	UK	Cohort study	Users of the COVID Symptom study app (vaccinated: n = 103,622; unvaccinated: n = 464,356)	16–99 years; Comirnaty: 54.5 years (SD: 14.3); Vaxzevria: 60.8 years (SD: 13.5); unvaccinated: 49.4 years (SD: 14.6)	Not reported	Comirnaty; Vaxzevria	Comirnaty: 45–59 days after dose 1; Vaxzevria: 21–44 days after dose 1	Comirnaty: 72% (63–79); Vaxzevria: 60% (49–68)	Not reported
Monge [14]; 15 Apr 2021^a^	Spain	Retrospective cohort study	LTCF inhabitants (n = 299,209)	Mean: 85.9 years	Not reported	99.8% Comirnaty	15–21 days after dose 1; ≥ 7 days after dose 2	51.0% (49.7–52.3)	81.2% (80.2–82)
Moustsen-Helms [15]; 9 Mar 2021^a^	Denmark	Retrospective cohort study	LTCF inhabitants (n = 39,040); HCW (n = 331,039)	LTCF median: 84 years (IQR: 77–90); HCW median: 47 years (IQR: 36–57)	Not reported	LTCF: > 99% Comirnaty HCW: 89% Comirnaty	> 14 days after dose 1; > 7 days after dose 2	HCW: 17% (4–28), LTCF inhabitants: 21% (− 11–44)	HCW: 90% (82–95) LTCF inhabitants: 64% (14–84)
Pawlowski [16]; 27 Feb 2021^a^	US	Matched case–control study	62,138 persons tested at Mayo Clinics	≥ 18 years	Not reported	Comirnaty; Moderna	> 36 days after dose 1; 1–2 weeks after dose 2	83.4% (60.2–94.3)	88.7% (68.4–97.1%)
Pritchard [29]; 23 Apr 2021^a^	UK	Prospective cohort study	Population-based (n = 373,402)	≥ 16 years	Alpha (B.1.1.7); Non-Alpha	Comirnaty; Vaxzevria	≥ 21 days after dose 1 and dose 2 (only Comirnaty)	Alpha: 66% (OR: 0.34 (0.28–0.41)) Non-Alpha: 71% (OR: 0.29 (0.16–0.51))	Alpha: 78% (OR: 0.22; (0.15–0.32)) Non-Alpha: 82% (OR: 0.18 (0.06–0.51))
Shrotri [17]; 7 Apr 2021^a^	UK	Cohort study	LTCF inhabitants (n = 10,412)	Mean: 86 years	Mainly Alpha (B.1.1.7)	Comirnaty (33%); Vaxzevria (67%)	35–48 days after dose 1	62% (23–81) (HR: 0.38 (0.19–0.77)) Comirnaty: 65% (HR: 0.35 (0.17–0.71) Vaxzevria: 68% (HR 0.32 (0.15–0.66))	Not reported
Swift [30]; 26 Apr 2021^a^	US	Retrospective cohort study	HCW at Mayo Clinics (n = 71,152)	Median: 41 years	Not reported	Comirnaty; Moderna	> 14 days after dose 1 and ≤ 14 days from dose 2; > 14 days after dose 2	Comirnaty: 78.1% (71.1–82.0) Moderna: 91.2% (80.6- 96.1)	Comirnaty: 96.8% (95.3–97.8) Moderna: 98.6% (90.1–99.8)
Tang [31]; 6 May 2021	US	Cohort study	HCW (n = 5,217)	Adults	Not reported	Comirnaty	≥ 12 days after dose 1 and before dose 2; ≥ 7 days after dose 2	58% (IRR: 0.42 (0.26–0.70))	96% (IRR: 0.04 (0.02–0.09))
Thompson [18]; 2 Apr 2021	US	Prospective cohort study	HCW, first responders, other essential and frontline workers (n = 3,950)	≥ 18 years	Not reported	Comirnaty (62.7%); Moderna (29.6%); unknown mRNA vaccine (7.7%)	≥ 14 days after dose 1 and dose 2	80% (59–90)	90% (68–97)

aIRR: adjusted incidence rate ratio; CI: confidence interval; COVID-19: coronavirus disease; HCW: healthcare workers; HR: hazard ratio; IQR: interquartile range; IRR: incidence rate ratio; LTCF: long-term care facility; RCT: randomised controlled trial; SARS-CoV-2: severe acute respiratory syndrome coronavirus 2; SD: standard deviation; UK: United Kingdom; US: United States.

^a^ Preprint.

One-dose efficacy/effectiveness was investigated in 24 studies (Table 1) and estimates ranged from 16.9% to 91.2%, with the majority of estimates ranging between 60% and 70%. The VE was lower in older (e.g. long-term care facility inhabitants) than in younger participants (e.g. healthcare workers). However, age-related effects could not be assessed in a number of studies since subgroup data were not reported. Vaccine type and study design did not appear to have an impact on VE estimates.

In 17 of 26 studies, VE was reported after the second dose. Estimates ranged between 61.7% and 98.6%. One study found an incidence reduction of 99% [10]. The majority of estimates ranged from 80% to 90%. Again, VE estimates were not affected by participant age, vaccine type and/or study design (see Supplement Part S4 for exact definitions of outcomes).

### Prevention of asymptomatic infection

Seven of 30 studies investigated VE against asymptomatic SARS-CoV-2 infections [11,19-22,31,32] (Table 2). With the exception of one study that had a multicentre design with study centres in the US, Brazil and South Africa, the remainder were performed in single centres in Israel (n = 3), the UK (n = 1) and the US (n = 2). Studies included between 5,217 and more than 300,000 participants, with three of them including healthcare workers only. Only the multicentre study investigating COVID-19 vaccine Janssen was an RCT [22]. The other studies had a cohort design and investigated Comirnaty or Comirnaty and COVID-19 vaccine Moderna using either hospital, insurance or surveillance data. In five of these six studies, VE against asymptomatic infection after one dose of Comirnaty or COVID-19 vaccine Moderna ranged from 36% to 79%. Five cohort studies also analysed VE against asymptomatic infection after a second dose and reported VE estimates between 80% and 94%. For the single-dose regimen of COVID-19 vaccine Janssen, VE against asymptomatic infections was 74% in the RCT [22].

**Table 2:** Efficacy and effectiveness of COVID-19 vaccines against asymptomatic SARS-CoV-2 infection, 1 January–14 May 2021 (n = 7)

Study	Country	Study design	Study population (n)	Age	Circulating variant	Vaccine	Time point of analysis after vaccine dose	Adjusted vaccine efficacy/effectiveness (95% CI)	
								After dose 1	After dose 2
Angel [32]; 6 May2021	Israel	Retrospective cohort study	HCW (n = 6,710)	Mean (SD) all: 44.3 (12.5) years; vaccinated: 44.8 (12.5) years; unvaccinated: 40.7 (11.7) years	Not reported	Comirnaty	7-28 days after dose 1; > 7 days after dose 2; > 21 days after dose 2	36% (IRR: 0.64; 0.31–1.51)	86% (IRR: 0.14 (0.07–0.31)) 94% (IRR: 0.02 (0–0.06))
EMA Assessment report COVID-19 vaccine Janssen [22]; 11 Mar 2021	Multicentre (incl. US, Brazil, South Africa)	RCT (phase 3-licensure trial)	Vaccine group (n = 19,306); placebo group (n = 19,178)	≥ 18 years	Beta (B.1.351) (); Zeta (P.2); D614G-carrying ‘WT/ref’	COVID-19 vaccine Janssen	> 28 days after vaccination	74% (27.9–92.4)	Not applicable
Haas [11]; 24 Mar 2021	Israel	Cohort study	Surveillance data (national); n = 202,684 SARS-CoV-2 infections; n = 102,012 non-vaccinated	≥ 16 years	Alpha (B.1.1.7) (94.5%)	Comirnaty	≥ 7 days after dose 2; ≥ 14 days after dose 2	Not reported	91.5% (90.7–92.2) 93.8% (93.3–94.2)
Jones [21]; 8 Apr 2021	UK	Retrospective cohort study	HCW (n = 8,776)	Adults	Mainly Alpha (B.1.1.7 )	Comirnaty	≥ 12 days after dose 1	75%^a^	Not reported
Tande [19]; 10 Mar 2021^b^	US	Retrospective cohort study	Population-based; patients at Mayo Clinics (n = 39,156)	≥ 18 years, Mean: 54,2 years (SD 19.7)	Not reported	Comirnaty; Moderna	> 10 days after dose 1; > 0 days after dose 2	79% (RR: 0.21 (0.12–0.37)) Comirnaty only: 79% (RR = 0.21 (0.11–0.38))	80% (RR: 0.20 (0.09–0.44)) Comirnaty only: 80% (RR: 0.20 (0.09–0.44))
Tang [31]; 6 May2021	US	Cohort study	HCW (n = 5,217)	Adults	Not reported	Comirnaty	≥ 12 days after dose 1 and before dose 2; ≥ 7 days after dose 2	42% (IRR: 0.58 (0.30–1.13))	90% (IRR: 0.10 (0.04–0.22))
Zacay [20]; 3 Mar 2021^b^	Israel	Retrospective cohort study	Insurance members (n = 6,286)	≥ 16 years	Mainly Alpha, (B.1.1.7) also Beta (B.1.351 )	Comirnaty	≥ 14 days after dose 1; ≥ 7 days after dose 2	61% (49–71)	89% (82–94)

CI: confidence interval; COVID-19: coronavirus disease; HCW: healthcare workers; IRR: incidence rate ratio; RCT: randomised controlled trial; RR: risk ratio; SARS-CoV-2: severe acute respiratory syndrome coronavirus 2; SD: standard deviation; UK: United Kingdom; US: United States.

^a^Own calculation.

^b^Preprint.

### Risk of bias

Risk of bias was low in one RCT [22]. In the other RCT [8], we detected some concerns due to different vaccine dosages being analysed. Of the 28 non-randomised studies, risk of bias was critical in four studies which did not adjust for confounders and reported unadjusted estimates. In a further two non-randomised studies, risk of bias was considered to be serious because adjustment for confounders was inappropriate. Besides one study with unclear risk of bias, all remaining studies had moderate risk of bias, mainly due to possible residual confounding (see Supplement Part S5 for details).

## Discussion

These interim results of a living systematic review show that after completed course the EMA-approved COVID-19 vaccines have a VE of 80% to 90% in preventing SARS-CoV2 infections, including asymptomatic ones. We found some indication that VE estimates are not reduced in cases infected with variant of concern (VOC) Alpha (Phylogenetic Assignment of Named Global Outbreak (Pango) lineage designation B.1.1.7), however these results should be interpreted with caution. VE against infection can also be regarded as an indicator of how well a vaccine prevents transmission. In addition, studies suggest that persons who become SARS-CoV-2-positive despite vaccination had a shorter duration of virus shedding and lower viral load [8].

Some of our methodological limitations stem from the rapidly changing publication landscape of COVID-19 vaccine studies. In particular, non-randomised studies on real-world effectiveness are continuously and frequently published on preprint servers. Although we systematically searched seven preprint servers, additional studies could have been published on other servers or websites that we did not capture. Moreover, it has to be considered that these studies did not undergo peer review and should therefore be considered with caution. A recent study reported that some final journal publications of COVID-19 studies differ to a certain extent from versions which were previously published on pre-print servers [33]. A further limitation is the small number of countries where the studies were performed, including the possibility that some studies, in particular those from Israel and the US, might have analysed partly overlapping study populations. The majority of studies included were conducted in persons vaccinated with Comirnaty. At the time point of data cut for this interim analysis, only limited information was available on VOCs other than Alpha. Meanwhile, some studies have been published indicating reduced effectiveness against infections with VOC Delta for Comirnaty and Vaxzevria, whereas effectiveness against hospitalisation was unchanged, as compared to VOC Alpha [34,35]. 

## Conclusion

Results of this living systematic review imply that COVID-19 vaccines are highly effective in preventing SARS-CoV-2 infections, including those which are asymptomatic. From a public health perspective, it can be concluded that fully vaccinated persons might in some instances still become PCR-positive for SARS-CoV-2 but only play a minor role in the transmission of SARS-CoV-2.

## Supplementary Data

665000 Supplement
