# Supplementary material for: Efficacy and effectiveness of COVID-19 vaccines against SARS-CoV-2 infection: interim results of a living systematic review, 1 January to 14 May 2021
Source: Euro Surveill. 2021 Jul 15;26(28):2100563. doi: 10.2807/1560-7917.ES.2021.26.28.2100563 (PMC8284046; doi:10.2807/1560-7917.ES.2021.26.28.2100563)
Supplement: Supplement [file 21-00563_HARDER_Supplement.pdf]

Supplementary material

*This supplementary material is hosted by Eurosurveillance as supporting information alongside the article "***Efficacy and effectiveness of COVID-19 vaccines against SARS-CoV-2 infection: first interim analysis of a living systematic review***", on behalf of the authors, who remain responsible for the accuracy and appropriateness of the content. The same standards for ethics, copyright, attributions and permissions as for the article apply. Supplements are not edited by Eurosurveillance and the journal is not responsible for the maintenance of any links or email addresses provided therein.*

Supplement part 1: PRISMA checklist

Section/topic	#	Checklist item	Reported on page #
**TITLE**			
Title	1	Identify the report as a systematic review, meta-analysis, or both.	1
**ABSTRACT**			
Structured summary	2	Provide a structured summary including, as applicable: background; objectives; data sources; study eligibility criteria, participants, and interventions; study appraisal and synthesis methods; results; limitations; conclusions and implications of key findings; systematic review registration number.	2
**INTRODUCTION**			
Rationale	3	Describe the rationale for the review in the context of what is already known.	3
Objectives	4	Provide an explicit statement of questions being addressed with reference to participants, interventions, comparisons, outcomes, and study design (PICOS).	3 and Supplement part 2
**METHODS**			
Protocol and registration	5	Indicate if a review protocol exists, if and where it can be accessed (e.g., Web address), and, if available, provide registration information including registration number.	3
Eligibility criteria	6	Specify study characteristics (e.g., PICOS, length of follow-up) and report characteristics (e.g., years considered, language, publication status) used as criteria for eligibility, giving rationale.	3
Information sources	7	Describe all information sources (e.g., databases with dates of coverage, contact with study authors to identify additional studies) in the search and date last searched.	3 and Supplement part 3
Search	8	Present full electronic search strategy for at least one database, including any limits used, such that it could be repeated.	Supplement part 3
Study selection	9	State the process for selecting studies (i.e., screening, eligibility, included in systematic review, and, if applicable, included in the meta-analysis).	3
Data collection process	10	Describe method of data extraction from reports (e.g., piloted forms, independently, in duplicate) and any processes for obtaining and confirming data from investigators.	3-4

Data items	11	List and define all variables for which data were sought (e.g., PICOS, funding sources) and any assumptions and simplifications made.	4
Risk of bias in individual studies	12	Describe methods used for assessing risk of bias of individual studies (including specification of whether this was done at the study or outcome level), and how this information is to be used in any data synthesis.	4
Summary measures	13	State the principal summary measures (e.g., risk ratio, difference in means).	4
Synthesis of results	14	Describe the methods of handling data and combining results of studies, if done, including measures of consistency (e.g., I^2) for each meta-analysis.	4

Section/topic	#	Checklist item	Reported on page #
Risk of bias across studies	15	Specify any assessment of risk of bias that may affect the cumulative evidence (e.g., publication bias, selective reporting within studies).	4
Additional analyses	16	Describe methods of additional analyses (e.g., sensitivity or subgroup analyses, meta-regression), if done, indicating which were pre-specified.	Not applicable
**RESULTS**			
Study selection	17	Give numbers of studies screened, assessed for eligibility, and included in the review, with reasons for exclusions at each stage, ideally with a flow diagram.	4 and Figure
Study characteristics	18	For each study, present characteristics for which data were extracted (e.g., study size, PICOS, follow-up period) and provide the citations.	4-5, Table 1 and 2
Risk of bias within studies	19	Present data on risk of bias of each study and, if available, any outcome level assessment (see item 12).	Supplement part 5
Results of individual studies	20	For all outcomes considered (benefits or harms), present, for each study: (a) simple summary data for each intervention group (b) effect estimates and confidence intervals, ideally with a forest plot.	Table 1 and 2
Synthesis of results	21	Present results of each meta-analysis done, including confidence intervals and measures of consistency.	Not applicable
Risk of bias across studies	22	Present results of any assessment of risk of bias across studies (see Item 15).	Not applicable
Additional analysis	23	Give results of additional analyses, if done (e.g., sensitivity or subgroup analyses, meta-regression [see Item 16]).	Not applicable

**DISCUSSION**			
Summary of evidence	24	Summarize the main findings including the strength of evidence for each main outcome; consider their relevance to key groups (e.g., healthcare providers, users, and policy makers).	5
Limitations	25	Discuss limitations at study and outcome level (e.g., risk of bias), and at review-level (e.g., incomplete retrieval of identified research, reporting bias).	5
Conclusions	26	Provide a general interpretation of the results in the context of other evidence, and implications for future research.	5
**FUNDING**			
Funding	27	Describe sources of funding for the systematic review and other support (e.g., supply of data); role of funders for the systematic review.	6

From: Moher D, Liberati A, Tetzlaff J, Altman DG, The PRISMA Group (2009). Preferred Reporting Items for Systematic Reviews and Meta-Analyses: The PRISMA Statement. PLoS Med 6(7): e1000097. doi:10.1371/journal.pmed1000097

Supplement part 2: PICO question

Participants/population: Male and female participants of all age groups

Intervention: Any vaccine against COVID-19 which has been approved for use in the European Union (or will be approved soon), including complete and incomplete dosing schedules

Comparators/control: placebo, no vaccination or a vaccine not directed against COVID-19 (active comparator), but also including head-to-head trials directly comparing different vaccines against COVID-19

Outcomes: 1. Efficacy and effectiveness-related outcomes: SARS-CoV2 infection (PCR-confirmed); hospitalisation due to COVID-19 (PCR-confirmed); ICU admission due to COVID-19 (PCR-confirmed); intubation and oxygen supply due to COVID-19 (PCR-confirmed); death due to COVID-19 (PCR-confirmed).
2. Safety-related outcomes: local reactions; systemic events; severe adverse events; enhanced COVID-19 disease; adverse events of special interest (AESI), including solicited and unsolicited events

Supplement part 3: Search strategy

The following searches will be combined with the terms "vaccin*" and "immuniz*" and the brand names of the approved vaccines.

Search Syntax PubMed 1:
("Severe Acute Respiratory Syndrome Coronavirus 2" [Supplementary Concept] OR "COVID-19" [Supplementary Concept] OR "COVID 19 diagnostic testing" [Supplementary Concept] OR "COVID 19 drug treatment" [Supplementary Concept] OR "COVID 19 serotherapy"[Supplementary Concept] OR "COVID 19 vaccine" [Supplementary Concept] OR "Severe Acute Respiratory Syndrome Coronavirus 2"[tiab] OR ncov*[tiab] OR COVID*[tiab] OR sars-cov-2[tiab] OR "sars cov 2"[tiab] OR "SARS Coronavirus 2"[tiab] OR "Severe Acute Respiratory Syndrome CoV 2"[tiab] OR "Wuhan coronavirus"[tiab] OR "Wuhan seafood market pneumonia virus"[tiab] OR "SARS2"[tiab] OR "2019-nCoV"[tiab] OR "hcov-19"[tiab] OR „novel 2019 coronavirus“[tiab] OR "2019 novel coronavirus*"[tiab] OR „novel coronavirus 2019*“[tiab] OR "2019 novel human coronavirus*"[tiab] OR „human coronavirus 2019“[tiab] OR "coronavirus disease-19"[tiab] OR "corona virus disease-19"[tiab] OR "coronavirus disease 2019"[tiab] OR "corona virus disease 2019"[tiab] OR "2019 coronavirus disease"[tiab] OR "2019 corona virus disease"[tiab] OR „novel coronavirus disease 2019“[tiab] OR „novel coronavirus infection 2019“[tiab] OR "new coronavirus*"[tiab] OR "coronavirus outbreak"[tiab] OR "coronavirus epidemic"[tiab] OR "coronavirus pandemic"[tiab] OR "pandemic of coronavirus"[tiab]) AND ("2019/12/01"[PDAT] : "2099/12/31"[PDAT])

Search Syntax PubMed 2:
("wuhan"[tiab] or china[tiab] or hubei[tiab]) AND ("Severe Acute Respiratory Syndrome Coronavirus 2"[Supplementary Concept] OR "COVID-19" [Supplementary Concept] OR "COVID 19 diagnostic testing"[Supplementary Concept] OR "COVID 19 drug treatment"[Supplementary Concept] OR "COVID 19 serotherapy"[Supplementary Concept] OR "COVID 19 vaccine"[Supplementary Concept] OR "coronavirus*"[tiab] OR "corona virus*"[tiab] OR ncov[tiab] OR COVID*[tiab] OR sars*[tiab])

Search Syntax Embase 1:
('severe acute respiratory syndrome coronavirus 2':ti,ab OR 'severe acute respiratory syndrome coronavirus 2'/exp OR 'COVID 19'/exp OR ncov*:ti,ab OR COVID*:ti,ab OR 'sars cov 2':ti,ab OR 'sars-cov-2':ti,ab OR 'sars coronavirus 2':ti,ab OR 'sars coronavirus 2'/exp OR 'severe acute respiratory syndrome cov 2':ti,ab OR 'wuhan coronavirus':ti,ab OR 'wuhan seafood market pneumonia virus':ti,ab OR sars2:ti,ab OR '2019-ncov':ti,ab OR 'hcov-19':ti,ab OR 'novel 2019 coronavirus':ti,ab OR '2019 novel coronavirus*':ti,ab OR 'novel coronavirus 2019'/exp OR '2019 novel human coronavirus*':ti,ab OR 'human coronavirus 2019':ti,ab OR 'coronavirus disease-19':ti,ab OR 'corona virus disease-19':ti,ab OR 'coronavirus disease 2019':ti,ab OR 'coronavirus disease 2019'/exp OR 'corona virus disease 2019':ti,ab OR '2019 coronavirus disease':ti,ab OR 'novel coronavirus 2019*':ti,ab OR 'novel coronavirus disease 2019':ti,ab OR 'novel coronavirus infection 2019':ti,ab OR '2019 corona virus disease':ti,ab OR 'new coronavirus*':ti,ab OR 'coronavirus outbreak':ti,ab OR 'coronavirus epidemic':ti,ab OR 'coronavirus pandemic':ti,ab OR 'pandemic of coronavirus':ti,ab OR 'severe acute respiratory syndrome coronavirus 2 vaccine'/exp OR 'COVID 19 vaccine'/exp) AND 2020:py

Search Syntax Embase 2:
(wuhan:ti,ab OR china:ti,ab OR hubei:ti,ab) AND ('severe acute respiratory syndrome coronavirus 2':ti,ab OR 'severe acute respiratory syndrome coronavirus 2'/exp OR 'severe acute respiratory

syndrome coronavirus 2' OR 'COVID*':ti,ab OR 'COVID 19'/exp OR 'COVID 19' OR coronavirus*:ti,ab OR 'corona virus*':ti,ab OR ncov:ti,ab OR COVID*:ti,ab OR sars*:ti,ab OR 'sars coronavirus 2'/exp)

Manual search in ArRvix, BioRvix, ChemRvix, MedRvix, Preprints.org, ResearchSquare und SSRN

Supplement part 4: Outcome definitions in the included studies on efficacy and effectiveness of COVID-19 vaccines against SARS-CoV-2 infection (symptomatic and asymptomatic)

**Study (reference)**	**Outcome definition**
Abu-Raddad (27)	any documented infection (persons found positive by polymerasechain-reaction (PCR) testing for severe acute respiratory syndrome coronavirus 2 (SARS-CoV-2))
Amit (7)	positive SARS-CoV-2 PCR
Andrejko (8)	positive molecular test result for SARS-CoV-2 infection
Björk (28)	first positive SARS-CoV2 test result
Britton (9)	any positive PCR- or antigen-based SARS-CoV-2 test result
Chodick (10)	at least one record of primary positive SARS-CoV-2 PCR test
Corchado-Garcia (29)	positive SARS-CoV-2 test
Dagan (11)	documented SARS-CoV-2 infection confirmed by positive PCR test
EMA- *Assessment report* COVID-19 Vaccine Janssen (26)	first occurrence of SARS-CoV-2 infection (serologically and/or molecularly confirmed)
Emary (12)	any NAAT positive infection (consisting of primary symptomatic cases, non-primary symptomatic cases (those with other symptoms such as nausea or diarrhea), asymptomatic cases, and cases for which symptoms were unknown)
Fabiani (30)	SARS-CoV-2 infection, based on a positive antigenic test confirmed by RT-PCR on the same day
Glampson (13)	first positive swab for all individuals (lateral flow test results were excluded)
Guijarro (14)	SARS-CoV-2 positive antigen or PCR from nasal swabs

Haas (15)	all SARS-CoV-2 infections (symptomatic and asymptomatic)
Hall (16)	PCR confirmed SARS-CoV-2 infection
Lumley (17)	any PCR-positive result (i.e. either symptomatic or asymptomatic)
Mason (31)	SARS-CoV-2 positive PCR test result
Menni (32)	SARS-CoV-2 test result
Monge (18)	documented SARS-CoV-2 infection
Moustsen-Helms (19)	laboratory confirmed SARS-CoV-2 infection (RT-PCR)
Pawlowski (20)	tested positive for SARSCoV-2 by PCR
Pritchard (33)	first new positive infection episodes
Shrotri (21)	first positive PCR test, indicating SARS-CoV-2 infection
Swift (34)	positive molecular assay for SARS-CoV-2
Tang (35)	Any positive test result
Thompson (22)	PCR-confirmed infection

Supplement part 5: Risk of bias assessments

Risk of bias in randomized controlled trials

Study	Bias arising from the randomization process	Bias due to deviations from the intended interventions	Bias due to missing outcome data	Bias in measurement of the outcome	Bias in selection of reported result	Summary
EMA Janssen	low	low	low	low	low	low
Emary	low	some concerns [1]	low	low	low	some concerns

[1] some participants received different dosages of the vaccine (low dose)

Risk of bias in non-randomized studies

Study	Bias due to confounding	Bias in selection of participants into the study/analysis	Bias in classification of interventions	Bias due to deviations from intended interventions	Bias due to missing data	Bias in measurement of outcomes	Bias in selection of reported result	Summary
Abu-Raddad	moderate [1]	low	low	low	low	low	low	moderate
Amit	serious [2]	low	low	low	low	low	low	serious
Andrejko	moderate [1]	low	moderate [3]	low	low	low	low	moderate
Angel	moderate [4]	low	low	low	low	low	low	moderate

Björk	moderate [4]	low	low	low	low	low	low	moderate
Britton	critical [5]	low	low	low	low	low	low	critical
Chodick	moderate [4]	low	low	low	low	low	low	moderate
Corchado-Garcia	moderate [4]	low	low	low	low	low	low	moderate
Dagan	moderate [4]	low	low	low	low	low	low	moderate
Fabiani	moderate [4]	low	low	low	low	low	low	moderate
Glampson	moderate [4]	low	low	low	low	low	low	moderate
Guijarro [6]	NA	NA	NA	NA	NA	NA	NA	unclear
Haas	moderate [4]	low	low	low	low	low	low	moderate
Hall	moderate [4]	low	low	low	low	low	low	moderate
Jones	critical [5]	low	low	low	low	low	low	critical
Lumley	moderate [4]	low	low	low	low	moderate [7]	low	moderate
Mason	moderate [4]	low	low	low	low	low	low	moderate
Menni	moderate [4]	moderate [8]	moderate [3]	low	low	moderate [9]	low	moderate
Monge	moderate [4]	low	low	low	low	low	low	moderate
Moustsen-Helms	serious [2]	low	low	low	low	low	low	serious
Pawlowski	moderate [4]	low	low	low	low	low	low	moderate

Pritchard	moderate [4]	low	moderate [10]	low	low	low	low	moderate
Shrotri	moderate [4]	low	low	low	low	low	moderate [11]	moderate
Swift	moderate [4]	low	low	low	low	low	low	moderate
Tande	moderate [4]	low	low	low	low	moderate [12]	low	moderate
Tang	critical [5]	low	low	low	low	low	low	critical
Thompson	moderate [4]	low	moderate [10]	low	low	low	low	moderate
Zacay	critical [5]	low	low	low	low	low	low	critical

[1] test-negative design; [2] only adjusted for community exposure rates; [3] self-reported vaccination status; [4] adjusted estimates reported, but residual confounding possible; [5] no confounder-adjusted estimates reported; [6] Due to study design (cohort study with elements of an interrupted time series), ROBINS-I could not be applied. According to the EPOC tool for interrupted time series, three out of seven domains would be judged as high risk of bias and two domains as unclear risk of bias. Therefore, the study was assessed here at unclear risk of bias. [7] some participants underwent symptomatic testing, but extent unknown; [8] selection bias cannot be excluded since App users might be special population; [9] self-reported outcome; [10] partly self-reported vaccination status; [11] participants with two doses excluded and results not reported; [12] unclear whether symptomatic persons were always excluded
